# Effectiveness of an Educational Intervention with Guidelines from the Total Acceleration of Postoperative Recovery Project (ACERTO) in Gynecology

**DOI:** 10.1055/s-0043-1772484

**Published:** 2023-11-29

**Authors:** Juliana Marques Marra, Isabela Corrêa Samper, Laura Aparecida Xavier de Abreu, Rafaela Pereira Anelvoi, Maria Gabriela Baumgarten Kuster Uyeda, Marair Gracio Ferreira Sartori, Gisele Vissoci Marquini

**Affiliations:** 1 Universidade Federal de Uberlândia, Uberlândia, MG, Brazil; 2 Escola Paulista de Enfermagem, Universidade Federal de São Paulo, SP, Brazil

**Keywords:** preoperative care, perioperative care, surgical procedures in gynecology, enhanced recovery after surgery, cuidados pré-operatórios, cuidados perioperatórios, procedimentos cirúrgicos em ginecologia, recuperação melhorada após a cirurgia

## Abstract

**Objective** To evaluate the effectiveness of an educational intervention among gynecologists about recommendations of the Total Acceleration of Postoperative Recovery (ACERTO, in the Portuguese acronym) project derived from the solid foundations of Enhanced Recovery After Surgery (ERAS) guidelines to optimize hospital care for surgical-gynecological patients.

**Methods** Educational intervention through monthly 1-hour long meetings (3 months), with the application of an objective questionnaire about specific knowledge of the ACERTO project between before and after educational intervention phases, for gynecologists, after approval by the ethics committee and signature of informed consent by participants, in a federal university hospital.

**Results** Among the 25 gynecologists who agreed to participate, the educational intervention could be effective with a statistically significant difference between the phases before and after the intervention for the main recommendations of the ACERTO project, such as abbreviation of preoperative fasting ( *p* = 0.006), venous thromboembolism prophylaxis ( *p* = 0.024), knowledge and replication of ACERTO ( *p* = 0.034), and multimodal analgesia ( *p* = 0.021).

**Conclusion** An educational intervention, through clinical meetings with exposition and discussion of the recommendations of the ACERTO project based on the ERAS protocol can be effective for the knowledge and possibility of practical application of the main measures, such as abbreviation of preoperative fasting, multimodal analgesia, and prophylaxis of thrombosis among gynecologists.

## Resumo

**Objetivo** Avaliar a efetividade de uma intervenção educativa entre ginecologistas de um hospital universitário a fim de capacitar o conhecimento científico das recomendações do projeto Aceleração da Recuperação Total Pós *-* operatória ACERTO, derivado das bases sólidas do protocolo *Enhanced Recovery After Surgery* (ERAS) para otimizar a assistência hospitalar de pacientes cirúrgico-ginecológicas.

**Métodos** Intervenção educativa por meio de reuniões mensais por 3 meses, com duração de 1 hora, com aplicação de questionário objetivo com questões de conhecimentos específicos do projeto ACERTO com fases antes e depois da intervenção, para profissionais ginecologistas, após aprovação do comitê de ética em pesquisas (CEP) e assinatura do termo de consentimento livre e esclarecido (TCLE) pelos participantes.

**Resultados** Dentre os 25 ginecologistas que aceitaram participar, a intervenção educativa se mostrou eficaz com diferença estatisticamente significante entre as fases antes e depois da intervenção para as principais recomendações do projeto ACERTO, como abreviação de jejum pré-operatório ( *p* = 0.006), profilaxia de tromboembolismo venoso ( *p* = 0.024), conhecimento e replicação do conhecimento do ACERTO ( *p* = 0.006) e analgesia multimodal ( *p* = 0.006).

**Conclusão** Uma intervenção educativa, por meio de reuniões clínicas com exposição e discussão das recomendações do projeto ACERTO baseadas em evidências e derivadas do ERAS é eficaz para o conhecimento e possibilidade de aplicação prática de medidas como abreviação de jejum pré-operatório, analgesia multimodal e profilaxia de trombose entre ginecologistas.

## Introduction

The Acceleration of Total Postoperative Recovery (ACERTO) project is a program that aims to optimize the postoperative recovery time in patients who undergo surgical procedures. 1 The program brings together pre, intra, and postoperative measures that favor the surgical outcome in several areas such as abdominal, bariatric, pediatric, orthopedic, and also in the gynecological area. Derived from the solid foundations of the Enhanced Recovery After Surgery (ERAS), the ACERTO project, in Brazil and other Latin American countries, prioritizes evidence-based medicine in addition to being an educational program. 1 2 3

Currently, several medical societies, including the International Federation of Gynecology and Obstetrics (FIGO), 4 American College of Obstetricians and Gynecologists (ACOG), 5 the European Society for Clinical Nutrition and Metabolism (ESPEN), 6 and the Brazilian Federation of Gynecology and Obstetrics (FEBRASGO), 7 among others, are engaged in the development of clinical guidelines and protocols that improve the quality of perioperative care through ERAS. Mastery of the surgical technique is important; however, perioperative care favors positive surgical results, in addition to maximizing recovery, minimizing unpleasant symptoms such as pain, reducing complications such as thrombosis, and decreasing hospitalization time and hospital costs. 1 2 3 4 5 6 7

The term ACERTO was an acronym developed in Latin America for ERAS with the purpose of favoring the understanding and adherence of the ERAS recommendations with local adaptations. 1 The ACERTO project, just like ERAS guidelines, focuses on clear information to the patient about the surgery, abbreviation of preoperative fasting, early postsurgical patient ambulation, thrombosis prophylaxis, early oral refeeding, optimization of the use of drains and probes to avoid unnecessary use, hyperhydration prevention, and facilitatation of analgesia to stimulate the recovery of patients undergoing surgical treatment. 1 2 3 4 5 6 7

Despite the scientific support of the ACERTO Project's recommendations, few hospitals or services apply its guidelines in practice due to lack of knowledge and adherence of health professionals. Given this scenario, the objective of the present study was to evaluate the effectiveness of an educational intervention in the gynecology department of a university hospital on the main recommendations of the ACERTO project to foster the knowledge and adherence of professionals in the practical applicability of standardized measures, such as the abbreviation of the preoperative fasting, prophylaxis for thromboembolic events, and multimodal and humanized analgesia approach to enable postoperative recovery in gynecological surgeries.

## Methods

**Fig. 1:**
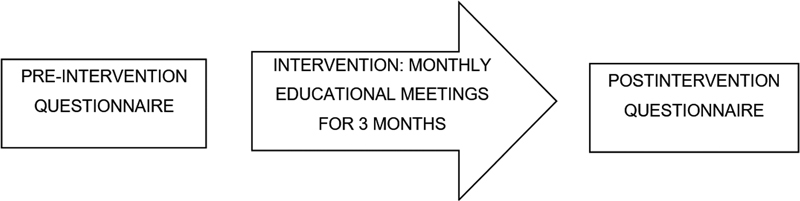
Schematic design of the study.

The study is quantitative and prospective, with a before and after design of an educational intervention ( Figure 1 ). It was carried out only after approval by the Research Ethics Committee (REC) of Universidade Federal de Uberlândia (UFU), with a favorable opinion CAAE: 57830922.5.0000.5152, in the department of gynecology of the Hospital de Clínicas da Universidade Federal de Uberlândia (HCUFU), a regional university hospital managed by Empresa Brasileira de Serviços Hospitalares (EBSERH). The methodology consisted of an educational intervention to medical professionals invited to participate in the study, from the area of gynecology, through scientific meetings that addressed the main recommendations of the ACERTO project in perioperative care for surgical-gynecological patients. The inclusion criteria were gynecologists from the gynecology sector of HCUFU who agreed to participate in the research and signed the informed consent form (ICF). The exclusion criteria were: Not accepting to participate in the research, not signing the ICF, or not belonging to the HCUFU gynecology sector.

All teaching gynecologists, residents and preceptors were invited, and only those who voluntarily met the inclusion criteria, participated in the sample. After clarifying the project and signing the ICF, they filled out a knowledge assessment questionnaire of the main recommendations of the ACERTO project (attached). The questionnaire was standardized with 30 questions, 10 of which were YES or NO, 10 with simple justification and 10 multiple-choice, with a completion time of approximately 5 to 10 minutes on knowledge of abbreviation of preoperative fasting, prophylaxis for thromboembolic events, knowledge about multimodal analgesia and surgical metabolism. This questionnaire was approved by the REC of the institution as a data collection instrument, delivered to the participating gynecologists, with confidentiality of the names of the participants, with only the sample number identification for later construction of the database, containing questions about knowledge, applicability, and development of the ACERTO project, as well as the advantages for the patient in the postoperative period and the safety in applying it. The same questionnaire was applied after the educational intervention.

- Application of the printed questionnaire and delivery to the gynecologists before the educational intervention or preintervention (PRE) phase on the recommendations of the ACERTO project.
- Educational explanation about the benefits of the ACERTO project parameters to research participants. The educational intervention consisted of monthly meetings of 30 to 40 minutesfor 3 months, with at least 20 minutes of open time for discussions and suggestions to encourage adherence to effective measures to accelerate the patients' recovery.
- Application of the printed questionnaire and delivery to the gynecologists after the educational intervention, or postintervention phase (POST), on the recommendations of the ACERTO project ( Figure 1 ).

To calculate the sample size, the following formula was used, described by Fontenelle et al. 8 where: zα/2 = Alpha error value (two-tailed); zβ = Beta error value; s = standard deviation; d = Minimum difference to be detected. The sample size was determined so that it could identify, with 95% confidence (α error = 0.05), a difference, if any, of at least 5 participants between the before and after groups who would possibly adhere or not to the ACERTO project, among the averages. The power of the test was considered to be 80% (error β = 0.20). Therefore, through this calculation, a sample of at least 20 participants from the gynecology department was suggested. This would be the minimum number of participants for the survey not to be terminated. The Chi-square or Fisher test was used to analyze categorical variables. Results were expressed as mean, followed by standard deviation (SD) or mean standard error or median. Data were analyzed using the IBM SPSS Statistics for Windows, version 20.0 software (IBM Corp., Armonk, HY, USA), with license granted to the department of mathematics at UFU. The data were analyzed quantitatively, with data protection, academic secrecy, until the publication of the results, with the objective of evaluating whether the educational intervention proved to be effective in obtaining a significant response regarding the characterization of the scientific knowledge of gynecologists about the ACERTO project derived from of ERAS at HCUFU.

## Results

The data analyzed after applying the methodology described above are expressed below. Table 1 shows a homogeneous sample between phases, with a total of 25 participants from 38 professional gynecologists in the department (65.78%), the majority being female, with a mean age of 27 years and less than 5 years since graduation. According to the data in Table 2 , there was effectiveness of the educational intervention carried out to promote knowledge of the ACERTO project among the analyzed sample, with a statistically significant difference between the phases of before, or preintervention (PRE), and after, or postintervention (POST). Knowledge was consolidated in the POST phase in relation to the abbreviation of fasting, one of the priority guidelines of the ACERTO project, with clarification that the ingestion of clear liquid, enriched with carbohydrate and protein, without residues, up to 2 hours before surgery, does not predispose to the risk of pulmonary aspiration of gastric contents (Mendelson syndrome). 9 The effectiveness of the educational intervention was confirmed by the willingness to reproduce and apply knowledge in practice among gynecologists, as analyzed in Table 2 .

**Table 1:** Descriptive Statistics: Demographic data of the sample

	PHASE	Mean	Standart deviation Min	Max
**AGE**	**PRE**	27.1	2.90	25 34
	**POST**	27.1	2.90	25 34
GENDER*		**Female N (%)**	**Male N (%)**	
	**PRE**	20 (80.0%)	5 (20.0%)	
	**POST**	20 (80.0%)	5 (20.0%)	
**TIME OF GRADUATION (y)**		**Less than 5y N (%)**	**Greater than 5y N (%)**	
	**PRE**	22 (88.0%)	3 (12.0%)	
	**POST**	22 (88.0%)	3 (12.0%)	

Abbreviation: y, years.

Chi-square: *p* = 0.248.

*self-definition of gender identity.

**Table 2:** Knowledge of the ACERTO project and/or Mendelson syndrome and the ability to replicate and apply it in care practice

Knowledge of the ACERTO project and/or Mendelson syndrome			
PHASE	YES	NO	TOTAL
**PRE**	12 (48.0%)	13 (52.0%)	25 (100.0%)
**POST**	25 (100.0%)	0 (0.0%)	25 (100.0%)
**Total**	12 (75.0%)	4 (25.0%)	25 (100.0%)
Chi-square: *p* = 0.021			
**The ability to replicate and apply it in care practice.**			
**PHASE**	**YES**	**NO**	**TOTAL**
**PRE**	4 (16.0%)	21 (84.0%)	25 (100.0%)
**POST**	25 (100.0%)	0 (0.0%)	25 (100.0%)
**Total**	29 (58.0%)	21 (42.0%)	50 (100.0%)

Chi-square: *p* = 0.034.

According to the data in Tables 3 and 4 , the educational intervention on prophylaxis recommendations for venous thromboembolism (VTE) aligned with the ACERTO project and ERAS among the evaluated gynecologists. These measures proved to be effective between the PRE and POST phases, both through criteria for mechanical and drug prophylaxis ( *p* = 0.024) as well as encouraging early walking in the postoperative period ( *p* = 0.006).

**Table 3:** Cited criteria for deep venous thromboembolism prophylaxis in the pre and postintervention phases

Criteria described	PHASE	
	PRE	POST
	N (%)	N (%)
Caprini BMI, history of DVT	3 (12.0%) 1 (4.0%)	23 (92.0%) 0
Previous history, pathologies, surgical size	1 (4.0%)	0
Did not specify	13 (52.0%)	1 (4.0%)
Did not answer	7 (28.0%)	1 (4.0%)
TOTAL	25 (100%)	25 (100%)

Abbreviations: BMI, body mass index; DVT, deep venous thromboembolism.

Chi-square: *p* = 0.024.

**Table 4:** Comparison of early walking recommendation between the pre and post intervention phases

Time to stimulate early ambulation	PHASE	
	PRE	POST
	N (%)	N (%)
After recovery from spinal anesthesia	14 (56%)	22 (88.0%)
2 H	3 (12.0%)	0
4 H	1 (4.0%)	2 (8.0%)
6 H	1 (4.0%)	0
12 H	2 (8.0%)	0
Did not answer	4 (16%)	1 (4.0%)
TOTAL	25 (100.0%)	25 (100.0%)

Chi-square: *p* = 0.006.

According to the ACERTO project criteria, multimodal analgesia involves pharmacological and non-pharmacological measures to minimize pain. However, the use of opioids is not recommended due to side effects. According to the data in Table 5 , the educational intervention was effective in consolidating this knowledge among the studied sample.

**Table 5:** Prescription of opioids within multimodal analgesia

PHASE	YES	NO	TOTAL
PRE	13 (52.0%)	12 (48.0%)	25 (100.0%)
POST	0 (0.0%)	25 (100.0%)	25 (100.0%)
Total	13 (26.0%)	37 (74.0%)	50 (100.0%)

Chi-square: *p* = 0.021.

The same outcome can be observed in relation to the concept of abbreviation of fasting for solids and liquids PRE and POST intervention, in which gynecologists consolidated with statistical difference the recommendation that fasting for solids can be established for up to 6 hours and for liquids without residues up to 2 hours before surgery ( Table 6 ).

**Table 6:** Comparison between knowledge of preoperative fasting time for solids and liquids without residues before and after the intervention

**Preoperative fasting time for clear liquids without residues**	**PHASE**	
	**PRE**	**POST**
	**N (%)**	**N (%)**
2H	13 (52.0%)	25 (100%)
6 H	8 (32.0%)	0 (0.0%)
8H	3 (12.0%)	0 (0.0%)
Did not answer	1 (4.0%)	0 (0.0%)
**TOTAL**	25 (100.0%)	25 (100.0%)
**Preoperative fasting time for solids**	**PHASE**	
	**PRE**	**POST**
	**N (%)**	**N (%)**
6 H	8 (32.0%)	25 (100.0%)
12 H	16 (64.0%)	0 (0.0%)
Did not answer	1 (4.0%)	0 (0.0%)
**TOTAL**	25 (100.0%)	25 (100.0%)

Chi-square: *p* = 0.0098.

## Discussion

The results of the present study indicate that the perioperative care recommendations consolidated by the ERAS, despite being published and endorsed by several medical societies, are not always adhered to in practice, as observed in the statements of the questionnaire about such recommendations before a didactic explanation. On the other hand, the efforts to adapt the main recommendations such as abbreviation of preoperative fasting, prophylaxis of VTE, and stimulation of multimodal, as occurred with the ACERTO project, are commendable initiatives to bring evidence-based medicine from practice. 1 2 3 4 5 6 7 10

In addition to projects adapted to the local reality, efforts to produce scientific discussions, training courses or educational interventions, such as the methodology used in this study, can be effective in disseminating knowledge and adherence to medical approaches such as paradigm shifts. The process of changing behavior occurs progressively with individual response; however, education provides the basis for the security of the practical application of new technologies. 8 9 11 12 According to recent studies on ERAS recommendations, barriers to implementation were analyzed, and the authors concluded that successful implementation requires a multidisciplinary team, a willingness to change, and a clear understanding of the protocol. Additionally, the difficulty in accomplishing necessary compliance to all protocol items calls for new implementation strategies, such as educational intervention. 11 12 13

In perioperative care, an educational intervention provided greater knowledge and possible adherence to measures, such as abbreviation of preoperative fasting. 1 This measure promotes a positive modulation of insulin resistance, with a lower risk of rebound hyperglycemia in the postoperative period, with a lower chance of complications derived from a diabetogenic state provided by prolonged fasting. 12 13 14 15 Other authors have already demonstrated the positive modulation of trauma response metabolism by ingestion of clear liquid enriched with carbohydrate (ERAS) and added protein (ACERTO) up to 2 hours before gynecological surgery, without putting the patient at risk of pulmonary aspiration. 9 12 13 14 15 16 17 18 19 The understanding of the physiology of fasting abbreviation in the positive modulation of surgical metabolism favors adherence to this measure. 9 12 13 14 15 16 17 18

Regarding pain management, the ACERTO project, aligned with ERAS, does not recommend the prescription of intraoperative and postoperative opioid analgesics, due to possible side effects such as drowsiness, dizziness, nausea, vomiting, and even respiratory depression. In addition to these effects being unpleasant for the patient, they make it difficult to approach early refeeding and walking, so encouraged by both projects. 12 13 14 15 16

The ERAS and ACERTO protocols recommend the standardization of criteria for prescribing mechanical and/or drug prophylaxis according to Caprini's parameters, enshrined in the literature. However, before the educational intervention, such criteria were not established in the choice of antithrombotic therapy for the gynecological patient. 20 21

According to the data from the present study, the educational intervention can be effective in consolidating concepts about antithrombotic therapy in perioperative care, previously not very well established among the sample of gynecologists evaluated. In addition, the simple early ambulation, recommended by the ERAS protocol and the ACERTO project, sometimes, unfortunately, of poor reinforcement in practice, is one of the pillars for the prevention of VTE, one of the most feared surgical complications in surgical procedures. 20 21 22 23 24 25 26 27 28 29

In university hospitals with standardized management, where basic measures of the recommendations of the ACERTO project were implemented, the process took place in an educational way, with the adherence of professionals after scientific knowledge of the solid, practical and reliable bases of the ACERTO project, with satisfactory results both as an improvement in the assistance such as resource optimization. 1 In Latin America, these data were observed at Hospital Universitário Júlio Muller of Universidade Federal do Mato Grosso and Hospital de São Paulo of Escola Paulista de Medicina, among others. 1

Challenges to the ERAS implementation may be encountered in other countries, regardless of the level of socioeconomic development. Health services from Canada, point out that the development of training sessions for health care professionals across the continuum of care (preoperative and admissions, operating room and postanesthesia care unit, and postoperative care) and the encouragement of their feedback can start an ERAS program. 30

The authors point out that the initial results remain under follow-up to assess the long-term adherence to the perioperative measures discussed and accepted by the majority of the body of gynecologists.

The positive point of the present study refers to the most recommended topic of perioperative care according to evidence-based medicine, with a methodologically rigorous, prospective study of practical analysis of adherence to these recommendations. In addition, it demonstrates a possibility of reproducibility that is easy to perform in any hospital care service to optimize surgical results and reduce hospitalization time and consequent hospital costs. Educational intervention can be the basis for the practical application of evidence-based medicine. Taking standardized scientific knowledge of measures such as abbreviation of preoperative fasting, pulmonary thromboembolism (PTE prophylaxis, and multimodal analgesia to the professional who is in the service practice can make a difference in the quality of hospital care.

Another positive point was the approval of the project by the hospital management where the study was carried out with financial and scientific support to serve as a pilot for other areas in order to reduce complications, optimize hospitalizations and reduce hospital costs.

The main difficulty in the project was recruiting the sample, as these are professional gynecologists with a workload who showed up for meetings during off-duty hours, which limited the sample size. However, the authors believe that this fact was overcome due to the engagement of voluntary, spontaneous participants, without recruitment or call, favoring the execution of the project within the sufficient number of sample according to the initial statistical calculation. Thus, the educational intervention methodology can be stimulated and reproduced in other hospital services with the expectation of favorable results, as in the present study.

## Conclusion

Educational intervention for gynecology professionals can consolidate scientific concepts of perioperative care endorsed by medical societies worldwide. This simple, low-cost, and highly reproducible methodology can be encouraged in other hospital services to align evidence-based medicine such as the ERAS-derived ACERTO project, with perioperative care in practice. Therefore, it is concluded that the perioperative recommendations for accelerating the patient's recovery, such as abbreviation of preoperative fasting, multimodal analgesia, and prophylaxis of thrombosis, complement and consolidate, as a fitting, after an educational intervention to an exceptional and humane quality of evidence-based care in gynecological surgery. The authors point out that this educational project, despite addressing a set of standardized perioperative recommendations, does not overcome the individualized decision of conduct based on the free exercise of medicine.
